# Investigating Factors Influencing the National Cancer Screening Program among Older Individuals in Republic of Korea—Data from the Korea National Health and Nutrition Examination Survey VIII

**DOI:** 10.3390/healthcare12121237

**Published:** 2024-06-20

**Authors:** Seok Hwan Kim, Hyo Eun Park

**Affiliations:** 1 Department of Health Information, Dongguk University Wise Campus, 123 Dongdae-ro, Gyeongju-si 38066, Gyeongsangbuk-do, Republic of Korea; rabbitear7@hanmail.net; 2 Department of Nursing, Suwon Women’s University, 72 Onjeong-ro, Gwonseon-gu, Suwon-si 16632, Gyeonggi-do, Republic of Korea

**Keywords:** National Cancer Screening Program (NCSP), older individuals, public health education, health status, health behaviors

## Abstract

This study aims to determine the influencing factors of the participation of older individuals aged 65 years and above in South Korea’s National Cancer Screening Program (NCSP) using data from the eighth wave (2019–2021) of the Korea National Health and Nutrition Examination Survey (KNHANES VIII), and discuss potential problems and coping strategies. Variables were selected based on Andersen’s healthcare utilization model. “Participation in the NSCP” was considered the dependent variable, with independent variables including sociodemographic characteristics (sex, marital status, residence, education level, income level, economic activity, medical coverage type, and private insurance), health conditions (subjective health status, hypertension, and diabetes), and health behaviors (physical activity, monthly alcohol consumption, and current smoking status). The analysis revealed that higher participation rates correlated with being married, having an education level beyond elementary school, being employed, subscribing to private insurance, perceiving oneself as having average or poor health, engaging in physical activity, and not smoking. Sex, residence, income, medical coverage type, hypertension, diabetes, and monthly alcohol consumption were found to be insignificantly correlated. These findings underscore the importance of tailored promotion and health education for older individuals to boost NCSP participation rates, which could ultimately elevate public health standards.

## 1. Introduction

Korea’s aging rate is unparalleled globally. As of 2000, the older population (65 years and older) constituted 7.2% of the total population, marking Korea as an aged society. This figure rose to 14.9% by 2018 and is projected to reach 20.3% by 2025 [1]. In 2021, Korea’s life expectancy was 80.6 years for men and 86.6 years for women, surpassing the OECD average. However, the healthy life expectancy was 64.4 years in 2018, marking the transition into an era of prolonged illness alongside increased longevity. The rapid increase in average lifespan is causing various socioeconomic challenges and escalating national healthcare costs, encompassing significant social and financial burdens [2]. Consequently, as the focus shifts from merely sustaining life to ensuring a healthy old age, interest in health-related quality of life is gaining momentum [3].

To enhance the health-related quality of life among older individuals, it is crucial to reduce the prevalence of diseases and related mortality rates, necessitating regular health check-ups for early detection and timely treatment. Regular health screenings, serving as preventive medical services, can help reduce the escalating national healthcare costs [4]. Thanks to early diagnosis, early treatment can be administered at lower costs. This not only improves the course of diseases but also efficiently safeguards public health, making such screenings strongly advocated at both national and community levels [5]. Additionally, health screenings have benefits like the possibility of straightforward treatments from early disease detection, favorable prognoses, and lower healthcare expenses, while reassuring those who receive negative results in early screenings [6].

Since 2000, neoplasms (C00-D48) have overtaken circulatory system diseases (I00–I99) as the leading cause of death in South Korea [7]. The gap in mortality rate (deaths per 100,000 people) between neoplasms and circulatory system diseases, the first and second leading causes of death, respectively, has been widening, from 0.7 in 2000 to 34.1 in 2010, and 42.0 in 2020. This indicates the significant death toll attributable to neoplasms. Prevention strategies encompass regular dietary habits, physical activity, the avoidance of harmful substances such as tobacco and alcohol, and also regular cancer screenings, which serve as a viable method of prevention [8].

What is commonly referred to as cancer includes malignant neoplasms (C00–C97), essentially the range of neoplasms (C00–D48) excluding benign neoplasms (D10–D36). In Korea, the National Health Insurance Service administers national cancer screenings to combat these diseases [9]. These screenings target individuals who align with specified cancer types, screening frequencies, and age guidelines pursuant to Article 11(2) of the Cancer Control Act, as specified in Article 52 of the National Health Insurance Act. The yearly cancer screening participation rate for older individuals has evolved as follows: 61.8% in 2016, 66.1% in 2017, 68.0% in 2018, 69.0% in 2019, 69.6% in 2020, and 65.3% in 2021. With rates fluctuating between 60% and 70%, screening participation for those aged 65 and above has been constantly higher than the 56.6% rate for individuals aged 19 to 65 as of 2021 [7]. Nonetheless, to attain the Third National Health Promotion Plan’s objective of achieving an 80% cancer screening rate across the entire population by 2020, further efforts to enhance screening participation are required.

Enhancing the cancer screening rate among older individuals is crucial for improving their health-related quality of life, and reducing medical expenses. In Korea, an older individual who reaches an average life expectancy of 83 years has a 35.5% chance of developing cancer, meaning one of three older individuals will be affected by cancer [10]. The incidence of cancer in the older population accounts for 11.7%, nearly triple that of the 35–64 age group [10]. Moreover, the expenditure on cancer treatment for older individuals amounted to KRW 3.6527 trillion, accounting for 40% of the total healthcare costs and showing an upward trend [1]. This highlights a direct association between the rising number of older cancer patients and increasing healthcare expenses.

Higher cancer-related medical expenses represent a significant challenge globally, not just in Korea. This situation places substantial physical, emotional, and financial strains on individuals, families, communities, and healthcare infrastructures. Older individuals, particularly, experience over 16 times higher mortality rates from cancer compared with other age groups, underscoring the increased risk of cancer incidence and mortality with age [11]. Nevertheless, countries with advanced healthcare systems have noted improvements in cancer survival rates. These advancements are mainly attributed to the availability of early detection, quality treatment, and effective management of cancer survivors [8]. This highlights the importance of developing healthcare systems that can mitigate the cancer burden, especially among older individuals.

To effectively address the cancer burden among older individuals, it is imperative to understand the impact of the National Cancer Screening Program (NCSP) on early detection and treatment. Previous research has explored various influencing factors of NCSP participation, including sociodemographic characteristics (sex, marital status, place of residence, education level, income level, economic activity, medical coverage type, private insurance), health conditions (self-perceived health, hypertension, diabetes), and health behaviors (physical activity, monthly alcohol consumption, current smoking status) [2,12,13,14,15,16,17,18,19,20,21]. The subjects of these studies include individuals with disabilities [12,14]; those aged 40 and over [13]; individuals aged 50–75 in specific regions [15]; patients with cervical cancer [16], breast cancer [17], and colorectal cancer [18]; those above 30 years [19]; those aged 65 and over in specific regions [2,20]; and the middle-aged and older individuals in specific regions [21].

Despite the significant interest in cancer and the preparation for an aging society, which has generated extensive research on cancer screenings, the research on the factors influencing older individuals’ participation in South Korea’s NCSP is notably scant. Developing strategies to enhance cancer screening rates among older individuals is crucial for improving their health-related quality of life, and minimizing medical expenses. This necessity is underscored by the observed differences in the reasons for nonparticipation in the NCSP between those aged 65 and above, and those younger than 65 [22]. Addressing this gap requires a thorough analysis of the factors that could potentially increase cancer screening rates among older adults. It is imperative to identify significant factors among sociodemographic characteristics, health conditions, and health behaviors that impact cancer screening rates. This involves using samples that represent the entire population from the regularly conducted Korea National Health and Nutrition Examination Survey (KNHANES) to identify factors significantly affecting the cancer screening rates among older individuals.

Against this backdrop, this study aims to investigate the factors affecting the participation of older individuals in the NCSP, aiming to provide foundational data for the development of detailed interventions and policy initiatives. These efforts aim to enhance the health-related quality of life and reduce medical expenses for the older population.

## 2. Materials and Methods

### 2.1. Data Collection

We extracted data for this study from the 8th Korea National Health and Nutrition Examination Survey (KNHANES VIII) conducted during the period from 2019 to 2021. This survey applied a complex, stratified, multistage probability sampling design based on age, sex, and region to accurately represent the non-institutionalized civilian Korean population. We also employed a rolling sample survey. To ensure consistent and reliable performance and reduce bias in the interviews and surveys, the KNHANES VIII adopted a technical investigation team comprising a nurse, a nutritionist, and a health science major, whose investigative performance was regularly verified and maintained through education and field quality control (all of this information is available on the KNHANES homepage). Out of the survey population (n = 25,559) of KNHANES VIII (2019–2021), we excluded individuals who were older than 65 years (n = 5285). After eliminating samples with missing data (n = 1214), 4071 participants were analyzed.

The KNHANES VIII receives annual deliberation and approval from the Research Ethics Deliberation Committee of the Korea Centers for Disease Control and Prevention (KCDC), and all participants provided written consent. We submitted a data use plan to the KCDC and posted a written pledge on the KNHANES homepage. Consequently, we received approval to use the data.

### 2.2. Selection of Variables

We employed the Andersen Model of Health Service Utilization to determine factors influencing older individuals’ participation in the NCSP. The Andersen model, established as a framework for predicting health service utilization [23,24], organizes individual characteristics affecting healthcare service utilization into predisposing, enabling, and need factors. Its efficacy in categorizing these factors has been confirmed by extensive research, proving its adequacy in predicting behaviors through the comprehensive management of both internal and external individual factors [25].

Drawing from the Andersen model and leveraging the KNHANES data, we selected variables to elucidate their influence on the older individuals’ participation in the NCSP, grouped into three categories: (1) predisposing factors (sociodemographic variables; sex, married status, education level), (2) enabling factors, including economic resources (household income, type of medical coverage, economic activity, private insurance) and social support (presence of a spouse), and (3) need factors, covering health conditions (subjective health perception, hypertension, and diabetes) and health behaviors (physical activity, alcohol consumption, and smoking status). The selection of healthcare utilization types was sourced from examining the NCSP screening rate among older individuals, culminating in the choice of 15 distinct items.

### 2.3. Definition of Variables

#### 2.3.1. National Cancer Screening Program (NCSP)

Trained interviewers collected responses regarding the NCSP from all participants. The questionnaire conformed to a yes/no response design, “Have you received cancer screening in the last two years?”.

#### 2.3.2. Sociodemographic and Lifestyle Variables

Trained interviewers asked all the participants about their sociodemographic and lifestyle variables. Education level was categorized into two groups using the criterion of middle school graduates or higher. Household income was calculated by standardizing monthly income according to the number of family members (monthly income/√number of family members).

Employment status, health insurance coverage, and subscriptions to private health insurance were assessed using the following items: “Have you worked for more than 1 h for pay, or for more than 18 h as an unpaid family worker in the past week?”—No (0)/Yes (1). “Which health insurance are you covered by?”—Medical Aid (0)/Health insurance (1). “Have you subscribed to any private health insurance offered by insurance companies, such as cancer insurance, cardiovascular disease insurance, or accident coverage insurance?”—No (0)/Yes (1).

Participants were categorized into two groups using the criterion of alcohol consumed within one month of the interview [26]. Smoking status was categorized into two groups according to respondents’ answers on the self-report questionnaire: current smoker or not. Based on the responses to the modified form of the International Physical Activity Questionnaire for Koreans, individuals were regarded as regular physical exercisers if they performed moderate exercise more than 5 times per week for over 30 min per session or performed vigorous exercise more than 3 times per week for over 20 min per session [27]. A face-to-face interview was used to obtain data about their place of residence (rural versus urban) and having a spouse.

#### 2.3.3. Biochemical Measurements

Trained staff members in the Division of Chronic Disease Surveillance under the Korea Centers for Disease Control and Prevention and the Korean Ministry of Health and Welfare took the physical measurements of the participants. A standard mercury sphygmomanometer (Baumanometer; W.A. Baum Co., Inc., Copiague, NY, USA) was used to measure blood pressure. Systolic blood pressure and diastolic blood pressure were measured twice at 5 min intervals, and the average values were used for the analysis. To measure concentrations of serum fasting plasma glucose, a blood sample was collected from the antecubital vein of each participant after fasting for >8 h. Blood samples were analyzed within 24 h of transportation. The levels of serum fasting plasma glucose were measured using a Hitachi Automatic Analyzer 7600 (Hitachi, Tokyo, Japan) by enzymatic methods using commercially available kits (Daiichi, Tokyo, Japan). Serum 25-hydroxyvitamin D levels were measured using a gamma counter (1470 Wizard; PerkinElmer, Wallac, Turku, Finland) by radioimmunoassay (RIA) using a 25-hydroxyvitamin D 125 I RIA kit (DiaSorin, Stillwater, MN, USA).

Chronic disease was defined according to the American Heart Association/National Heart, Lung, and Blood Institute Scientific Statement criteria for Asians [28]. According to these criteria, three or more of the following criteria must be fulfilled to be diagnosed: blood pressure ≥130/85 mm Hg, use of antihypertensive medication and fasting blood glucose ≥100 mg/dL, or current use of anti-diabetes medication. Diabetes was diagnosed when fasting blood sugar was >126 mg/dL or when the individual was currently using anti-diabetic medications [29].

### 2.4. Statistical Analysis

Data analysis was performed using the SPSS ver. 27.0 (IBM Co., Armonk, NY, USA) with a *p*-value of less than 0.05 considered statistically significant. The analysis aimed to elucidate the general characteristics of the participants and to identify variables that influence participation in the NCSP.

First, a frequency analysis was performed to examine the participants’ sociodemographic characteristics, health conditions, and health behaviors.

Second, a chi-square analysis was conducted to investigate the rate of participation in the NCSP across different participant characteristic variables.

Third, a binary logistic regression analysis was applied to pinpoint factors that significantly affected the likelihood of participants engaging in the NCSP, based on the results of the chi-square analysis.

## 3. Results

### 3.1. Participants’ General Characteristics

Table 1 outlines the frequency distribution according to the general characteristics of the participants. Among those subject to the NCSP, 2819 individuals (69.2%) had been screened, while 1252 individuals (30.8%) had not. By sex, females (n = 2268, 55.7%) outnumbered males (n = 1803, 44.3%). Regarding marital status, 1268 individuals (31.1%) were unmarried, and 2803 individuals (68.9%) were married. Regarding place of residence, 1189 individuals (29.2%) lived in rural areas, and 2882 individuals (70.8%) lived in urban areas. Regarding education level, 2116 individuals (52.0%) had an elementary school education or less, 717 individuals (17.6%) had completed middle school, 800 individuals (19.7%) had completed high school, and 438 individuals (10.8%) had a college degree or higher. The most frequent income category was low (n = 1762, 43.3%), followed by lower-middle (n = 1200, 29.5%), upper-middle (n = 695, 17.1%), and high (n = 414, 10.2%). Regarding economic activity, there were 2505 unemployed individuals (61.5%) and 1566 employed individuals (38.5%). Regarding the type of medical coverage, 286 individuals (7.0%) were under Medical Aid, and 3785 individuals (93.0%) were covered by health insurance. Regarding private insurance, 1823 individuals (44.8%) were not subscribed, and 2248 individuals (55.2%) were subscribed. Subjective health status was rated as poor by 1104 individuals (27.1%), average by 1987 individuals (48.8%), and good by 980 individuals (24.1%). Hypertension was reported by 2545 individuals (62.5%), and was absent among 1526 individuals (37.5%), while diabetes was reported by 1227 individuals (30.1%) and was absent among 2844 individuals (69.9%). Physical activity was not practiced by 3441 individuals (84.5%) and was practiced by 630 individuals (15.5%). The monthly drinking rate in the past year was less than one drink per month for 2706 individuals (66.5%) and one drink or more per month for 1365 individuals (33.5%). In terms of current smoking status, 3706 individuals were non-smokers or past smokers (91.0%), and 365 individuals were current smokers (9.0%).

### 3.2. Cancer Screening Status According to the Participants’ Characteristics

Table 2 presents an analysis of the cancer screening rates based on participants’ characteristics. When analyzing sociodemographic factors, married individuals (n = 2050, 73.1%) had significantly higher participation rates in the NCSP than unmarried individuals (n = 769, 60.6%), with this difference being statistically significant (*p* < 0.001). Regarding education, the highest participation was noted among those with a college degree or higher (n = 345, 78.8%) (*p* < 0.001). Regarding income levels, the upper-middle income group showed the greatest participation rate (n = 528, 76.0%) (*p* < 0.001). Higher screening rates were observed in the following groups: the employed (n = 1146 individuals, 73.2%) compared with the unemployed (n = 1673, 66.8%) (*p* < 0.001); individuals covered by health insurance (n = 2657, 70.2%) compared with those receiving Medical Aid (n = 162, 56.6%) (*p* < 0.001); and those with private insurance (n = 1717, 76.4%) compared with those without (n = 1102, 60.4%) (*p* < 0.001). Regarding health conditions, participants without hypertension (n = 1086, 71.2%) had a slightly higher screening rate than those with hypertension (n = 1733, 68.1%) (*p* < 0.05). Concerning health behaviors, higher screening rates were observed among individuals engaged in physical activity (n = 1363, 72.3%) compared with the inactive (n = 1456, 66.6%) (*p* < 0.001), current drinkers (n = 988, 72.4%) compared with non-drinkers (n = 1831, 67.7%) (*p* < 0.01), and non-smokers (n = 2590, 69.9%) compared with current smokers (n = 229, 62.7%) (*p* < 0.01). However, factors such as sex, residence, subjective health status, and diabetes did not show statistically significant differences in screening rates.

### 3.3. Factors Influencing the Participants’ Cancer Screening Rates

The regression analysis conducted to explore factors influencing cancer screening among participants, as shown in Table 3, generated the following findings: regarding the sociodemographic characteristics, higher screening rates were observed among married individuals (1.526 times, *p* < 0.001) compared with unmarried individuals (*p* < 0.001); middle school graduates (1.326 times, *p* < 0.01), high school graduates (1.442 times, *p* < 0.001), and college graduates or higher (1.824 times, *p* < 0.001) compared with those with elementary education or less; the employed (1.227 times, *p* < 0.01) compared to the unemployed; and those with private insurance (1.813 times, *p* < 0.001) compared to those without. Regarding health conditions, higher screening rates were observed among participants who had reported their subjective health status as average (1.294 times, *p* < 0.05) or poor (1.266 times, *p* < 0.01) compared with good. Among health behaviors, participants who adhered to physical activity exhibited a 1.162 times higher cancer screening rate (*p* < 0.05) compared with those who did not. Similarly, non-smokers had a 1.566 times higher screening rate than current smokers (*p* < 0.001). However, factors such as sex, place of residence, income level, type of medical coverage, hypertension, diabetes, and monthly drinking rate did not show statistically significant differences.

## 4. Discussion

This study leverages data from the Korea National Health and Nutrition Examination Survey (KNHANES) to explore factors that affect older individuals’ participation in the National Cancer Screening Program (NCSP). The factors were grouped into sociodemographic characteristics, health conditions, and health behaviors. Through the analyses, we identified marital status, education level, economic activity, private insurance subscription, subjective health status, and current smoking status as significant determinants of cancer screening participation among older individuals. This study is significant in that it identifies key characteristics unique to the older population, setting them apart from the broader adult demographic. By doing so, it aims to furnish foundational data essential for developing practical intervention strategies and formulating policy recommendations. These initiatives are geared toward enhancing the health-related quality of life for older individuals and minimizing their medical expenses.

The study involved 4071 individuals aged 65 and over, with 2819 participants (69.2%) having undergone the NCSP in the last two years. The screening rate of participants was higher than the 67.8% reported by the National Health Insurance Service for 2020 [1], but lower than the 74.2% reported by the National Statistical Office for 2023 [30]. However, it did not reach the 80% NCSP target set by the National Health Promotion Plan HP2030, indicating a need for multifaceted efforts to improve screening rates.

Among the sociodemographic characteristics, marital status, education level, and economic activity were identified as the factors influencing NCSP participation. Regarding marital status, individuals with spouses demonstrated a 1.525 times higher rate of participation in the NCSP than unmarried individuals. Specifically, married participants had a participation rate of 68.4%, compared with 61.5% for widowed, 54.8% for divorced, and 47.3% for unmarried individuals. These figures translate to lower odds of participation for the widowed (OR = 0.59, 95% CI 0.58–0.59), divorced (OR = 0.56, 95% CI 0.55–0.56), and unmarried (OR = 0.47, 95% CI 0.47–0.48) individuals compared with their married counterparts. A study by Gram et al. [31] found that individuals with partners were five times more likely to participate in cancer screenings than those without, regardless of sex. This trend was particularly evident in colorectal cancer screenings, where those with partners demonstrated significantly higher participation rates [31]. Similar findings were reported in studies focusing on prostate, breast, and colorectal cancer screenings [32,33,34], reinforcing the notion that the support of a spouse or partner can enhance health monitoring and screening participation. In addition, in the UK, when living with a husband, understanding and participation in cervical cancer screening programs are high [35]. These observations suggest that family members, especially spouses, play a pivotal role in encouraging cancer screenings and providing the necessary social support for such health-promoting behaviors.

Analysis by education level revealed that the participation rates for cancer screenings were 1.326 times higher for middle school graduates, 1.442 times higher for high school graduates, and 1.824 times higher for college graduates or higher, compared with those with elementary education or lower. This aligns with findings from previous research, indicating a correlation between higher educational attainment and cancer screening rates [33,36,37,38,39]. And in a study of Europeans, those who received higher education were more likely to voluntarily participate in cancer screening programs [40,41]. This trend indicates increasing participation rates with higher education levels: 63.8% for those with elementary education or less, 72.9% for middle school graduates, 75.1% for high school graduates, and 78.8% for college graduates or higher. Considering that over half (52%) of the older population in Korea have an elementary education or less, enhancing cancer screening rates among less-educated older individuals emerges as a crucial goal. While higher education is associated with increased screening rates, it is considered a nonadjustable fixed variable. The positive correlation between higher education level and screening participation suggests that education influences knowledge, which subsequently affects health behaviors. Therefore, to motivate older Korean individuals with lower educational levels to actively participate in cancer screenings, boosting their health literacy is essential. This involves educating them on the importance of self-care and health awareness. Studies on rural older individuals and older cancer patients in Korea [42,43] and older individuals in China [44] show that health literacy positively influences cancer prevention behaviors and self-management, underscoring the importance of advocating for the NCSP as an effective cancer prevention strategy. Consequently, diverse educational strategies aimed at enhancing health literacy at both community and public healthcare levels are imperative to raise cancer screening rates among older individuals with lower educational attainment.

With respect to employment status, the NCSP participation rate was 1.227 times higher for employed individuals compared to unemployed ones, underscoring employment as a significant factor enhancing NCSP participation. This finding aligns with a previous study [45]. The screening rate among employed individuals stood at 73.2%, significantly higher than the 66.8% rate among the unemployed. The Ministry of Health and Welfare [46] notes an increasing trend in older individuals engaging in economic activities, with economically active seniors reporting higher life satisfaction [47]. This implies that employment fosters greater social interactions, which consequently positively influences perceptions toward cancer prevention. Employment significantly influences the frequency of regular health screenings among older individuals [48], with labor laws mandating health screenings for workers, suggesting that oversight by occupational health managers plays a role in screening participation [49]. However, the current 73.2% screening rate does not meet the national HP2030 target, underscoring the necessity for more health screening awareness and education in workplaces. Given that 61.5% of older Korean individuals are not employed, community health centers and primary healthcare facilities must adopt a multifaceted approach to boost their screening rates. Additionally, with various diseases and mobility challenges hindering some seniors from working [50], further research accounting for these factors is essential.

The analysis of the NCSP participation rate by private insurance status revealed that individuals with a private insurance subscription were 1.813 times more likely to participate than those without. This indicates that older Korean individuals with private insurance, in addition to national insurance, exhibit higher screening rates, aligning with findings from previous studies that reported higher screening rates among those with private insurance [51,52]. The study also found that individuals with private insurance typically have higher education levels and better financial standings [53], which suggests these individuals generally enjoy better health, fewer chronic diseases, and superior socioeconomic statuses compared with their uninsured counterparts. These findings, closely tied to socioeconomic status, demonstrate a consistent relationship with health screening rates, suggesting that improved economic conditions promote greater adherence to health screenings. The likelihood of using cancer screenings has been shown to increase with private insurance [54,55], presumably because it offsets out-of-pocket costs associated with early cancer detection [56]. In a healthcare system blending public social insurance with private healthcare insurance, the latter emerges as a crucial influencing factor of cancer screening rates by lowering economic barriers like out-of-pocket expenses, thereby improving economic accessibility.

Regarding health conditions, individuals with an average subjective health status participated in the NCSP at a rate 1.294 times higher, and those with poor health at a rate 1.266 times higher, than individuals who perceived their health as good. This suggests that those who consider their health less than ideal are more inclined to participate in cancer screenings, which is attributable to heightened concern for their health and a desire to preserve it. Regarding health behaviors, physically active individuals showed a 1.162 times higher participation rate than those who were inactive, likely because physical activity fosters social connections that facilitate access to cancer screening information. Additionally, non-smokers were found to have a 1.566 times higher screening rate than smokers. Given that smoking is a known risk factor for cancer, cerebrovascular, and coronary artery diseases, this underscores the critical need for regular cancer screenings among smokers. However, previous research [57,58,59] indicates that smokers are generally less inclined to seek health screenings. Furthermore, some research suggests that smoking correlates with cancer screenings only among women. The reluctance of long-term smokers to quit regardless of screening results, as well as the burden of being forced to quit smoking in case of adverse results, may deter them from screening. This aspect will need further investigation in future research. Additionally, incorporating cancer screening promotion and education into community centers and public health center smoking cessation programs could potentially increase screening rates among older smokers, drawing on successful models from similar initiatives.

However, factors such as sex, place of residence, income level, type of medical coverage, hypertension, diabetes, and monthly drinking rate were not statistically significant in influencing the NCSP participation rate. This study’s distinctive findings reveal that only factors like marital status, education level, economic activity, private insurance, subjective health status, physical activity, and current smoking status significantly influence participation, independent of the previously mentioned non-significant factors. This differentiation is attributable to the unique characteristics of the study’s participants, who were older individuals over the age of 65. Factors contributing to this phenomenon include the increasing likelihood of becoming solitary over time, lower education levels leading to a reduced exchange of information about screenings, and diminished access to medical services owing to a lack of economic activity, which consequently affects the ability to take out private insurance. Moreover, individuals who subjectively perceive themselves as healthy might be less inclined to engage in cancer screenings. Likewise, those not participating in regular physical activities may find themselves outside local community networks, resulting in limited access to information about cancer screenings. Additionally, smokers, potentially less concerned with health management, show a lower propensity for undergoing screenings. This tendency appears to be more pronounced with advancing age.

This study has limitations. First, cancer-related family history could not be investigated, and information on re-screenings was unavailable. More accurate results might have also been obtained by comparing and contrasting with an individual’s participation in cancer screenings besides the NCSP. And more variables such as an individual’s diet, job type, exposure to chemicals, and the environment can affect cancer health checkups, so it is necessary to study various factors affecting cancer health checkups through further research. In addition, cancer is considered necessary to classify and study according to various types of cancer health checkups such as lung cancer, breast cancer, liver cancer, and cervical cancer.

Despite these limitations, this study is significant because it was conducted by sampling older individuals nationwide with recent data, which can help future research related to cancer screenings for older individuals. For follow-up research, it is considered necessary to examine solitary older individuals.

This study identifies marital status, education level, economic activity, private insurance enrollment, subjective health status, physical activity, and current smoking rate as the influencing factors of the health screening participation rate of older individuals. Therefore, the following suggestions are made:

First, the National Health Insurance Service and cancer screening institutions should conduct targeted promotions to prevent older individuals from missing the cancer screening time window.

Second, health screening institutions should actively provide guidance to increase the participation of older individuals who are living alone owing to separation, divorce, or bereavement; those with a low education level; those not engaged in economic activities; and those without private insurance subscriptions in the NSCP.

Third, the government should emphasize the necessity of health screenings for those with a good subjective health status, and those who do not engage in physical activities.

Fourth, the community should inform smokers about the importance of participating in the NCSP and provide related health education.

Fifth, there are various types of cancer, and the factors affecting cancer health checkups may differ depending on gender, so further research is needed in the future.

## 5. Conclusions

This study aims to determine the influencing factors of the participation rates in the NCSP among older individuals and to propose measures to address any identified issues. This study is significant in that it targeted all elderly people in Korea using data from the eighth period of the National Health and Nutrition Survey.

The analysis results showed that among individuals aged 65 and older, factors such as marital status, education level, economic activity, private insurance subscription, subjective health status, physical activity, and smoking status significantly impacted NCSP participation. These factors were influential irrespective of gender, place of residence, income level, type of medical coverage, hypertension, diabetes, and monthly alcohol consumption.

Therefore, in order to increase the national cancer health examination rates for the elderly, publicity and health education for the elderly are necessary, and continuous guidance is required through various national approaches, accordingly boosting overall health screening coverage and ultimately improving national health standards.

## Figures and Tables

**Table 1:** General characteristics of participants.

Variable			n	%
Cancer Screening		Not examined	1252	30.8
		Examined	2819	69.2
Social·demographic characteristics	Sex	Female	2268	55.7
		Male	1803	44.3
	Married Status	Single	1268	31.1
		Married	2803	68.9
	Residence	Rural	1189	29.2
		Urban	2882	70.8
	Education level	Elementary school or less	2116	52.0
		Middle school graduate	717	17.6
		High school graduate	800	19.7
		College graduate or higher	438	10.8
	Income level	Low	1762	43.3
		Lower-middle	1200	29.5
		Upper-middle	695	17.1
		High	414	10.2
	Economic activity	Unemployed	2505	61.5
		Employed	1566	38.5
	Medical coverage type	Medical care	286	7.0
		Health Insurance	3785	93.0
	Private insurance	Not joined	1823	44.8
		Joined	2248	55.2
Health condition	Subjective health	Good	980	24.1
		Average	1987	48.8
		Bad	1104	27.1
	HTN	No	1526	37.5
		Yes	2545	62.5
	DM	No	2844	69.9
		Yes	1227	30.1
Health behavior	Physical activity	Do not practice	3441	84.5
		Practice	630	15.5
	Monthly drinking rate	Current drinker	1365	33.5
		Non-drinker	2706	66.5
	Current smoking status	Current smoker	365	9.0
		Non-smoker	3706	91.0
Total			4071	100.0

**Table 2:** National cancer screening rates according to characteristics of participants.

Variable			Cancer Screening		Total	χ^2^	*p*
			Not Examined	Examined			
Sociodemographic characteristics	Sex	Female	721 (31.8%)	1547 (68.2%)	2268 (100.0%)	2.581	0.108
		Male	531 (29.5%)	1272 (70.5%)	1803 (100.0%)		
	Married Status	Single	499 (39.4%)	769 (60.6%)	1268 (100.0%)	63.946	0.000
		Married	753 (26.9%)	2050 (73.1%)	2803 (100.0%)		
	Residence	Rural	387 (32.5%)	802 (67.5%)	1189 (100.0%)	2.539	0.111
		Urban	865 (30.0%)	2017 (70.0%)	2882 (100.0%)		
	Education level	Elementary school or less	766 (36.2%)	1350 (63.8%)	2116 (100.0%)	65.703	0.000
		Middle school graduate	194 (27.1%)	523 (72.9%)	717 (100.0%)		
		High school graduate	199 (24.9%)	601 (75.1%)	800 (100.0%)		
		College graduate or higher	93 (21.2%)	345 (78.8%)	438 (100.0%)		
	Income level	Low	649 (36.8%)	1113 (63.2%)	1762 (100.0%)	55.992	0.000
		Lower-middle	326 (27.2%)	874 (72.8%)	1200 (100.0%)		
		Upper-middle	167 (24.0%)	528 (76.0%)	695 (100.0%)		
		High	110 (26.6%)	304 (73.4%)	414 (100.0%)		
	Economic activity	Unemployed	832 (33.2%)	1673 (66.8%)	2505 (100.0%)	18.497	0.000
		Employed	420 (26.8%)	1146 (73.2%)	1566 (100.0%)		
	Medical coverage type	Medical care	124 (43.4%)	162 (56.6%)	286 (100.0%)	22.941	0.000
		Health Insurance	1128 (29.8%)	2657 (70.2%)	3785 (100.0%)		
	Private insurance	Not joined	721 (39.6%)	1102 (60.4%)	1823 (100.0%)	119.942	0.000
		Joined	531 (23.6%)	1717 (76.4%)	2248 (100.0%)		
Health condition	Subjective health	Good	299 (30.5%)	681 (69.5%)	980 (100.0%)	4.047	0.132
		Average	588 (29.6%)	1399 (70.4%)	1987 (100.0%)		
		Bad	365 (33.1%)	739 (66.9%)	1104 (100.0%)		
	HTN	No	440 (28.8%)	1086 (71.2%)	1526 (100.0%)	4.228	0.040
		Yes	812 (31.9%)	1733 (68.1%)	2545 (100.0%)		
	DM	No	863 (30.3%)	1981 (69.7%)	2844 (100.0%)	0.743	0.389
		Yes	389 (31.7%)	838 (68.3%)	1227 (100.0%)		
Health behavior	Physical activity	No	731 (33.4%)	1456 (66.6%)	2187 (100.0%)	15.827	0.000
		Yes	521 (27.7%)	1363 (72.3%)	1884 (100.0%)		
	Monthly drinking rate	Current drinker	377 (27.6%)	988 (72.4%)	1365 (100.0%)	9.478	0.002
		Non-drinker	875 (32.3%)	1831 (67.7%)	2706 (100.0%)		
	Current smoking status	Current smoker	136 (37.3%)	229 (62.7%)	365 (100.0%)	7.970	0.005
		Non-smoker	1116 (30.1%)	2590 (69.9%)	3706 (100.0%)		
Total			1252 (30.8%)	2819 (69.2%)	4071 (100.0%)		

**Table 3:** Factors influencing national cancer screening rates of participants.

Variable			B	*p*	OR	95% CI	
						Min.	Max.
Social·demographic characteristics	Sex	Female (ref.)	1.000				
		Male	−0.148	0.094	0.863	0.725	1.026
	Married Status	Single (ref.)	1.000				
		Married	0.423	0.000	1.526	1.299	1.792
	Residence	Rural (ref.)	1.000				
		Urban	−0.009	0.913	0.991	0.849	1.158
	Education level	Elementary school or less (ref.)	1.000	0.000			
		Middle school graduate	0.282	0.005	1.326	1.088	1.615
		High school graduate	0.366	0.000	1.442	1.178	1.765
		College graduate or higher	0.601	0.000	1.824	1.384	2.403
	Income level	Low (ref.)	1.000	0.135			
		Lower-middle	0.157	0.075	1.169	0.984	1.390
		Upper-middle	0.161	0.152	1.174	0.943	1.463
		High	−0.063	0.644	0.939	0.719	1.226
	Economic activity	Unemployed (ref.)	1.000				
		Employed	0.205	0.007	1.227	1.057	1.425
	Medical coverage type	Medical care (ref.)	1.000				
		Health Insurance	0.145	0.285	1.156	0.886	1.507
	Private insurance	Not joined (ref.)	1.000				
		Joined	0.595	0.000	1.813	1.564	2.101
Health condition	Subjective health	Good (ref.)	1.000	0.017			
		Average	0.258	0.012	1.294	1.058	1.583
		Bad	0.236	0.008	1.266	1.062	1.510
	HTN	No (ref.)	1.000				
		Yes	−0.060	0.420	0.942	0.815	1.089
	DM	No (ref.)	1.000				
		Yes	0.021	0.783	1.021	0.878	1.188
Health behavior	Physical activity	No (ref.)	1.000				
		Yes	0.150	0.037	1.162	1.009	1.338
	Monthly drinking rate	Current drinker (ref.)	1.000				
		Non-drinker	−0.132	0.114	0.877	0.744	1.032
	Current smoking status	Current smoker (ref.)	1.000				
		Non-smoker	0.448	0.000	1.566	1.227	1.997

## Data Availability

The datasets generated during and/or analyzed during the current study are available in the Korea Centers for Disease Control and Prevention repository [http://knhanes.cdc.go.kr]. The datasets generated during and/or analyzed during the current study are also available from the corresponding author upon reasonable request.
